# A Systematic Review and Meta-Analysis of Interventions Used to Reduce Exposure to House Dust and Their Effect on the Development and Severity of Asthma

**DOI:** 10.1289/ehp.10382

**Published:** 2007-09-25

**Authors:** Clare Macdonald, Anna Sternberg, Paul R. Hunter

**Affiliations:** School of Medicine, Health Policy and Practice, University of East Anglia, Norwich, United Kingdom

**Keywords:** asthma, atopy, children, house dust mite, meta-analysis, randomized control trial, systematic review

## Abstract

**Objectives:**

We assessed whether any household dust reduction intervention has the effect of increasing or decreasing the development or severity of atopic disease.

**Data sources:**

Electronic searches on household intervention and atopic disease were conducted in January 2007 in EMBASE, MEDLINE, and the Cochrane Central Register of Controlled Trials. No date or language restriction was placed on the literature search.

**Data extraction:**

We included randomized controlled trials comparing asthma outcomes in a household intervention group with either placebo intervention or no intervention.

**Data sysnthesis:**

Fourteen studies met the inclusion criteria. Eight recruited antenatally and measured development of atopic disease. Six recruited known atopic individuals and measured disease status change. Meta-analyses on the prevention studies found that the interventions made no difference to the onset of wheeze but made a significant reduction in physician-diagnosed asthma. Meta-analysis of lung function outcomes indicated no improvement due to the interventions but found a reduction in symptom days. Qualitatively, health care was used less in those receiving interventions. However, in one study that compared intervention, placebo, and control arms, the reduction in heath care use was similar in the placebo and intervention arms.

**Conclusions:**

This review suggests that there is not sufficient evidence to suggest implementing hygiene measures in an attempt to improve outcomes in existing atopic disease, but interventions from birth in those at high risk of atopy are useful in preventing diagnosed asthma but not parental-reported wheeze.

During the 1960s, hospital admission rates in England and Wales for childhood asthma steadily increased. This trend continued into the 1980s and began a steady decline in the 1990s, with no apparent explanation for this trend (van Schayck and Smit 2005). By 2000, annual admissions for asthma in England and Wales were 48 and 16 per 10,000 in children younger than 5 and those 5–14 years, respectively (National Statistics 2007). In 2004, asthma caused 1,266 deaths in England and Wales, 38 of which were children younger than 14 years, accounting for 2.9% of all deaths of 1- to 14-year-olds (National Statistics 2007). In the United States, the prevalence of self-reported asthma reached a peak of 60.5 per 1,000 population in children 0–4 years of age and 82.5 in children 5–14 years of age in 1995 and has since declined (Mannino et al. 2002).

A known risk factor for the development of atopic asthma is exposure and sensitisation to the house dust mite *Dermatophagoides pteronyssinus*. A meta-analysis of measures to reduce house dust mite exposure concluded there was no evidence to suggest implementing avoidance measures (Gøtzsche et al. 1998). However, the review did not include any prospective studies examining potential avoidance of atopic disease development. If the etiology of asthma and the role of household interventions in mitigating the disease can be more fully understood, there is an increased likelihood that asthma can be treated more appropriately and perhaps prevented.

Our primary objective was to assess whether any household intervention aimed at ameliorating exposure to house dust mite could reduce the incidence of asthma in high-risk children or reduce the severity of asthma in individuals already diagnosed with the disease.

## Data Sources

Potential studies on household intervention and asthma were identified from a series of electronic searches. Databases searched were the Cochrane Central Register of Controlled Trials (CENTRAL; http://www.mrw.interscience.wiley.com/cochrane/cochrane_clcentral_articles_fs.html), MEDLINE (http://gateway.ovid.com/), and EMBASE (http://www.embase.com/). Searches were conducted in week 2 of 2007, and no date or language restriction was placed on the literature search. The databases were searched with the following criteria: “hypersensitivity” [MeSH term] OR “hypersensitivity” [text word] OR “asthma” [MeSH term] OR “asthma” [text word] OR “eczema” [MeSH term] OR “eczema” [text word] OR “dermatitis atopic” [MeSH term] OR “dermatitis atopic” [text word] OR “allergy” [MeSH term] OR “allergy” [text word] AND “household” [MeSH term] OR “hygiene” [text word] OR “hygiene” [MeSH term] OR “hygiene” [text word] OR “animal domestic” [MeSH term] OR “domestic animal” [text word] OR “pets” [text word] OR “dust” AND “randomized controlled trials” [MeSH term] OR “randomized controlled trial” [text word] OR “randomized controlled trial” [text word] OR “RCT” [text word].

In addition, when close to completion, we performed a further search of articles searching only for recent articles and using the terms “dust” and “asthma” for 2006 onward. Also, the Institute of Scientific Information database of global conference proceedings (http://portal.isiknowledge.com/) was searched for abstracts of relevant presentations at scientific conferences for 2002 and later that had not yet been published as full articles.

Abstracts for all the publications identified by the search were reviewed and assessed for suitability for inclusion by two authors of the present article. Where disagreement occurred, a third assessor contributed to the final decision. Full copies of all suitable studies were obtained, and a further decision was made as to whether they fulfilled the search criteria. During the entire selection process, none of the authors were blinded to the source of the publication, its authors, or any other detail.

## Data Extraction

### Types of studies

All randomized trials that compared any household intervention to a control group with placebo (where practical) or no intervention were considered for this review.

### Types of participants

As atopic disease affects adults and children, no restriction was based on age, hence studies that had their own age restriction were considered. It was anticipated that there would be a mixture of studies recruiting in the antenatal period prospectively examining the development of atopic disease and those that recruited known atopic individuals.

Those studies recruiting subjects known to be atopic needed to have either a clinical diagnosis of asthma or positive skin prick test to a known trigger allergen. Prospective studies with antenatal recruitment had a first-degree relative with a clinical diagnosis of atopic disease or atopic disease confirmed on skin-prick testing. Studies would still be considered if they compared these individuals with lower-risk individuals, as long as there was a direct comparison between two groups of high-risk individuals receiving or not receiving a household intervention.

### Types of intervention

Inevitably, there would be a wide range of household interventions. Those considered for review were the following:

- Provision of allergen-impermeable bedding compared with no change in care or with placebo.
- Provision of household cleaning product or equipment (for example, high-efficiency vacuum cleaners) compared with no change or with the provision of placebo/alternative products.
- Education programs about allergen reduction measures compared with no additional educational input.
- Changes to home environment (for example, mold reduction and repairs to heating systems) compared with no change in home environment or with education only with no physical help.

### Types of outcome measures

Any outcome measure referring to the development, exacerbation, or severity of atopy was considered. We anticipated that many studies examining allergen reduction would measure allergen levels as a primary outcome. As this was not the aim of the present review, such studies would be included only if they also provided a measurement of atopic disease indicators. Types of outcome measures indicating the development of atopy were as follows: *a*) presence of wheeze (noted by parent or general practioner); *b*) diagnosis of asthma; *c*) prescribed asthma medication; and *d*) positive skin test to an aeroallergen.

Below are the types of outcome measures indicating the severity of atopy in studies in which participants were known to be atopic: *a*) severity of eczema; number of acute hospital/ clinic visits for asthma; *b*) combined asthma outcome [a measure based on treatment requirement and bronchial hyperreactivity to histamine used by Francis et al. (2003)]; *c*) lung function; *d*) peak flow; and *e*) diary days with chest tightness.

### Analysis

We methodologically assessed the studies meeting the inclusion criteria using a validated five-point scoring system (Jadad et al. 1996). This tool assesses quality of randomized controlled trials on the basis of the reported quality of randomization, blinding, and adequate descriptions of subject withdrawals and dropouts. The score is calculated according to whether the study is randomized and double blinded. Additional points are given based on the description of withdrawals and adequacy of description of the randomization and blindness. Data were extracted using a standard form adapted from a sample form provided by the Berkeley Systematic Reviews Group (2007). The main data extracted were duration of study, study size, nature of intervention, and outcome measures. The data were then entered into a Microsoft Excel (Microsoft Corp., Redmond, WA, USA) spreadsheet for analysis .

Meta-analysis was done using StatsDirect (StatsDirect 2007). For continuous outcomes such as peak flow or days of symptoms, an effects-size meta-analysis was performed. For dichotomous outcomes relative risk meta-analysis is reported. Given the diversity of interventions included, random-effects models were used. In addition, data were tested for bias and study heterogeneity.

## Data Synthesis

### Description of studies

The initial electronic search yielded 248 references. Duplicates (studies found by more than one database) were excluded and the remaining abstracts screened by C.R. and A.S. After the screening, 33 references were selected to be more thoroughly examined for potential inclusion. Eleven of these met the inclusion criteria for the present review. Three additional recent articles were identified using the search, bringing the total to 14 to meet the inclusion criteria.

The characteristics of the studies meeting the criteria for inclusion, including types of participants, duration of the study, and a summary of the intervention used are presented in Table 1.

One meeting abstract was identified that investigated the impact on symptoms of asthma, but this was a small study of only 44 children with asthma and no controls; thus, this was not analyzed further (Brugge et al. 2006).

### Methodological quality

The quality of reporting in the studies included was variable, with included studies all achieving either 2 or 3 with a mean Jadad score of 2.4. In some cases the study design was prohibitive of a blinding process, thus preventing the studies from reaching a score > 3 based on the Jadad criteria.

The population sizes in the studies identified ranged from 30 to 937 and the duration of follow-up ranged from 6 months to 7 years. Most studies did not provide placebo interventions for the control group and left the group with no change in their care. Three studies used placebo, two provided a nominal part of the intervention and one provided the same information and equipment after completion of the study period.

Separate analyses were performed of those studies measuring prevention and those assessing improvement.

### Prevention of asthma

Eight studies considered interventions to prevent asthma (Arshad et al. 2007; Becker et al. 2004; Chan-Yeung et al. 2000, 2005; Corver et al. 2006; Custovic et al. 2001; Marks et al. 2006; Schönberger et al. 2005). Three articles represented repeat analyses of the same cohort; thus, only the most recent report was included in the analysis (Becker et al. 2004; Chan-Yeung et al. 2000, 2005).

Five of the studies had comparable interventions, providing education about allergen exposure reduction as well as allergen reduction equipment (Arshad et al. 2007; Chan-Yeung et al. 2005; Corver et al. 2006; Custovic et al. 2001; Marks et al. 2006). The sixth did not implement any physical changes but provided a comprehensive education program on how to reduce newborns’ exposure to allergens (Schönberger et al. 2005).

**Figure 1:**
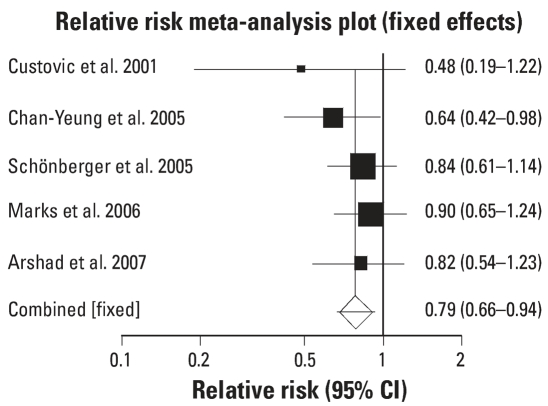
Meta-analysis [RR (95% CI)] of interventions to prevent physician-diagnosed asthma. Error bars indicate 95% CIs.

**Figure 2:**
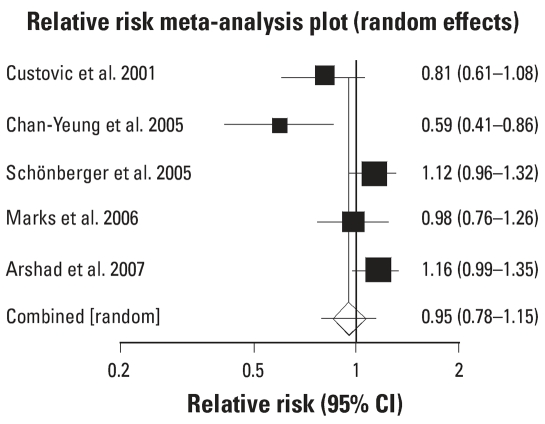
Meta-analysis [RR (95% CI)] of interventions to prevent parent-reported wheeze. Error bars indicate 95% CIs.

All six studies were analyzed for two outcome measures: physician-diagnosed asthma and parent-reported wheeze. From the data available in this outcome measure, a relative risk meta-analysis was performed. The forest plots are shown in Figures 1 and 2. All five studies that reported on physician-diagnosed asthma demonstrated a reduction in diagnosed asthma in the intervention arm by the end of the study period, although in only one was this statistically significant. Nevertheless, the pooled estimate showed a significant reduction [relative risk (RR) = 0.79; 95% confidence interval (CI) 0.66–0.94; *p* = 0.0093, fixed-effects model). There was no evidence of bias (0.091), and Cochran’s Q was not statistically significant (*p* = 0.626), supporting the use of the fixed-effects model.

For the five studies that reported on parent-reported wheeze, only one study showed a significant reduction in the intervention arm and two showed an actual increase. The combined effect of the interventions did not show a significant impact on parent-reported wheeze (RR = 0.95; 95% CI 0.78–1.15; *p* = 0.616). However, both bias (*p* = 0.005) and Cochran’s Q (*p* = 005) were statistically significant, indicating possible bias in the results and heterogeneity in the study outcomes.

### Improvement in already-diagnosed asthma

**Figure 3:**
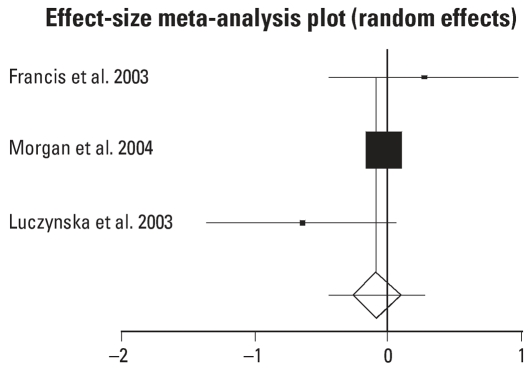
Effect of house dust reduction interventions on lung function. DL (DerSimonian-Laird) pooled effect size = −0.084057; 95% CI, −0.452474 to −0.28436. Error bars indicate 95% CIs.

Six of the identified studies were concerned with estimating the impact of house dust reduction on the severity of asthma (Carter et al. 2001; Francis et al. 2003; Kercsmar et al. 2006; Luczynska et al. 2003; Morgan et al. 2004; Williams et al. 2006). Many different outcome measures were used in these studies, and often the analyses presented for these outcome measures differed. Two outcome measures were used for meta-analysis. Three studies reported some form of a result of lung function such as FEV_1_ (forced expiratory volume in 1 sec) or peak flow (Francis et al. 2003; Luczynska et al. 2003; Morgan et al. 2004). For these three studies we conducted an effects-size meta-analysis (Figure 3). The pooled estimates of effects size did not show any significant impact of the intervention (−0.084; 95% CI, 0.452–0.284).

**Figure 4:**
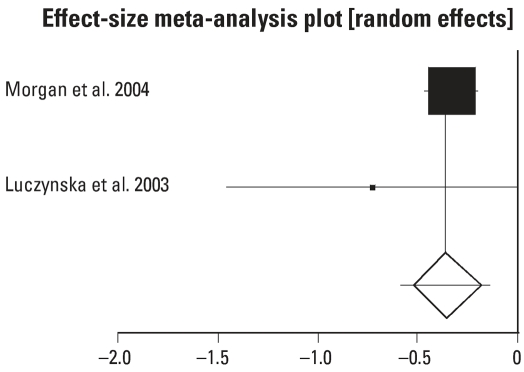
Effect of house dust reduction interventions on days ill with asthma. DL (DerSimonian-Laird) pooled effect size = −0.360796; 95% CI, −0.590095 to −0.131497. Error bars indicate 95% CIs.

There was sufficient information on only two studies to perform a meta-analysis for days with symptoms (Figure 4) (Luczynska et al. 2003; Morgan et al. 2004). The meta-analysis showed a significant reduction in days ill in the intervention group (−0.361; 95% CI, −0.590 to −0.131).

Several studies reported outcome measures for unplanned hospital or clinic visits; unfortunately the studies did not analyze the data similarly nor did they present their results in a form sufficient to allow a meta-analysis. However, one study worth mentioning used control, placebo, and intervention arms (Carter et al. 2001). The study identified a significant decrease in clinic attendances in the intervention and placebo arms but not in the control arms, suggesting that there is a strong placebo effect on subjective measures of asthma severity.

## Conclusions

One major difficulty in conducting this review and meta-analysis was the wide range of different outcome measures used and the ways in which such measures were analyzed and presented. This made extraction of data particularly difficult, especially for those studies investigating dust reduction interventions as a means of reducing the severity of asthma. It is necessary that there be standardization in reporting the results of studies of interventions aimed at reducing the severity of symptoms of asthma. A further issue was the relatively low Jadad score of only 2.4, which reflects the lack of blinding in almost all studies.

The results of the present meta-analysis suggest a significant reduction in physician-diagnosed asthma as a result of interventions to reduce exposure to house dust (RR = 0.74; 95% CI, 0.58–0.95). However, there was no significant effect on parent-reported wheeze (RR = 0.95; 95% CI, 0.78–1.15). This may suggest that reduced exposure to house dust prevents the more severe form of asthma but not the more common and milder forms. This is consistent with the observation that the majority of physician-diagnosed asthma cases in children are atopic, whereas many cases of parent-reported wheeze are often not atopic (Kaditis et al. 2007; Morais-Almeida et al. 2007). Indeed parents frequently err in reporting the presence of wheeze, both erroneously reporting its presence when it is not there and not recognizing wheeze when it is there (Cane et al. 2000; Cane and McKenzie 2001). Nevertheless, a 26% reduction in physician-diagnosed asthma would be a worthwhile benefit.

The results of the analyses on disease severity reduction were conflicting. The results of lung function did not show improvement (−0.084; 95% CI, −0.452 to 0.284), whereas the number of days ill, a more subjective estimate of severity, was reduced (−0.361; 95% CI, −0.590 to −0.131). Together, the findings of this review and the results of the meta-analysis give an uncertain and mixed estimate of the value of interventions aimed at reducing house dust on severity of asthma. The finding of a reduction in disease severity as measured by unplanned hospital and clinic attendances in both intervention and placebo arms in one study raises concerns that much of the impact of house dust reduction interventions may have a psychological rather than a direct effect (Carter et al. 2001). This is particularly the case given the lack of blinding in most studies.

A further explanation for the mixed results may be that the efficiency of the interventions at reducing exposure to antigens from house dust mites varied among studies. There is evidence from some studies that dust reduction measures may not be particularly effective (Carter et al. 2001), particularly for children living in poorer areas (Carter et al. 2001). However, Schönberger et al. (2004) assessed compliance with dust reduction procedures and found good compliance with antimite encasing advice but not with more demanding actions such as the use of smooth floor coverings in living room and nursery.

In conclusion, the evidence in favor of interventions aimed at reducing exposure to house dust for the prevention of physician-diagnosed asthma in high-risk children is strong. In this regard we consider the evidence to be stronger than the conclusions of other reviews (Semic Jusufagic et al. 2006). However, the impact of interventions appears not to affect parent-reported wheeze.

The evidence for house dust reduction in controlling the symptoms of asthma is currently weak, and it is not yet possible to advise on the general feasibility of this strategy in asthmatic children. One problem in determining the value of this intervention was the different outcome measure used and the way results were presented, which prevented a formal meta-analysis. Agreement is needed on appropriate standards for the conduct and analysis of future trials of environmental interventions to control the clinical severity of asthma.

## Correction

The last name of the first author has been changed from Russell in the original manuscript published online to Macdonald in the final version.

## Figures and Tables

**Table 1:** Randomized control studies of house dust reduction interventions on the prevention or control of asthma.^a^

Reference	Inclusion criteria	Duration of study	Control no.	Intervention no.	Control group intervention	Intervention group intervention	Measured prevention or improvement	Jadad score
Carter et al. 2001	Present on a database as being treated for asthma	12 months	35	34	No intervention	Placebo—allergen-permeable mattress and pillow covers, ineffective roach traps, no instructions	Improvement	2
				35		Allergen-impermeable mattress and pillow covers, roach bait, laundry instructions, and instructions about cleaning to control dust mites		
Custovic et al. 2001	Pregnancies with either: *a*) both parents atopic, positive to skin-prick test (high risk) and pets; *c*) both parents skin-prick test negative; no family history of atopy (low risk)	12 months	146	145	No intervention	Allergen-impermeable bedding covers fitted to parental bed, laundry instructions, and high-filtration vacuum cleaner, damp dusting.	Prevention	2
				161 168		Vinyl cushion flooring fitted in child’s bedroom, custom-made crib and cot mattresses, hot-washable soft toy, washing instructions		
Francis et al. 2003	Age between 18–65 years with a diagnosis of asthma and living with cat or dog	12 months	15	15	HEPA vacuum cleaners alone, vacuuming minutes 2× per week	Honeywell Envirocare HEPA cleaners in living room and bedroom and Dyson HEPA vacuum cleaners vacuuming minutes 2× per week	Improvement	2
Luczynska et al. 2003	Age 18–54 years with asthma diagnosis, taking inhaled steroids, sensitive to house dust mite	12 months	25	30	Sham allergen- proof bed covers	Allergen-proof bed covers	Improvement	2
Morgan et al. 2004	Age 5–11 years with an asthma diagnosis, asthma-related hospital admission and positive skin-prick test	12 months	468	469	No intervention	Providing child’s caretaker with knowledge, skills, motivation, equipment to perform environmental remediation with 5–7 home visits	Improvement	3
Schönberger et al. 2005	Asthma in at least mother, father, or siblings of unborn child	2 years	234	242	No intervention	Instruction from nurses on reducing mite allergens, pet allergens, food allergens, etc.	Prevention	3
Kercsmar et al. 2006	Age 2–17 years with symptomatic asthma for at least 3 months and hospital visit with asthma in past year	12 months	33	29	Information given improving home indoor air quality	Home remediation performed 4–5 months after study began, including cleaning, repairs, air conditioning, etc.	Improvement	3
Chan-Yeung et al. 2000	High risk for atopy—at least 1 first-degree relative with asthma or 2 second-degree with other allergic diseases identified in third trimester	12 months	267	278	No intervention	Mattress assessment, laundry instructions, benzyl benzoate application to carpets and furniture, counseled about pets, smoking cessation	Prevention	2
Becker et al. 2004	High risk for atopy—at least 1 first-degree relative with asthma or 2 second-degree with other allergic diseases identified in third trimester	2 years	267	278	No intervention	Mattress assessment, laundry instructions, benzyl benzoate application to carpets and furniture, counseled about pets, smoking cessation	Prevention	2
Chan-Yeung et al. 2005	High risk for atopy—at least 1 first-degree relative with asthma or 2 second-degree with other allergic diseases identified in third trimester	7 years	266	279	No intervention	Mattress assessment, laundry instructions, benzyl benzoate application to carpets and furniture, counseled about pets, smoking cessation	Prevention	2
Williams et al. 2006	English-speaking 5- to 12-year olds with asthma exacerbation presenting to ED living within the “Atlanta Empowerment Zone” (high level of poverty)	12 months	77	84	Same as intervention group, after the study period	Information and equipment to reduce allergen exposure: mattress encasing, laundry instructions, hydramethylnon gel, smoking advice, professional cleaning	Improvement	3
Marks et al. 2006	Prenatally identified high-risk individuals with at least 1 parent or sibling who had asthma or wheezing	5 years	308	308	Advice about allergen reduction. Provided poly- unsaturated oils and spreads and capsules low in ω-3 fatty acids	Impermeable bedding covering, laundry instruction, advice. Provided canola-based oils and spreads and capsules containing ω-3 fatty acids	Prevention	2
Arshad et al. 2007	Prenatally identified high-risk individuals (at least 2 family members with allergic disease)	8 years	62	58	No intervention	Elimination of dairy, fish, wheat, nuts, soya from diet, impermeable bedding covers, carpet, and upholstery treatment	Prevention	2
Corver et al. 2006	Prenatally identified high risk individuals (allergic mother)	4 years	394	416	Placebo bedding covers	Allergen-impermeable covers	Prevention	2
			472		No intervention			

Abbreviations: ED, emergency department; HEPA, high-efficiency particulate air.

a Articles by Chan-Yeung et al. (2000, 2005) and Becker et al. (2004), were analyses of the same cohort. Only the most recent articles were included in analyses.
